# Natural Variation in the Thermotolerance of Neural Function and Behavior due to a cGMP-Dependent Protein Kinase

**DOI:** 10.1371/journal.pone.0000773

**Published:** 2007-08-22

**Authors:** Ken Dawson-Scully, Gary A.B. Armstrong, Clement Kent, R. Meldrum Robertson, Marla B. Sokolowski

**Affiliations:** 1 University of Toronto, Department of Biology, Mississauga, Ontario, Canada; 2 Queen's University, Department of Biology, Kingston, Ontario, Canada; Freie Universitaet Berlin, Germany

## Abstract

Although it is acknowledged that genetic variation contributes to individual differences in thermotolerance, the specific genes and pathways involved and how they are modulated by the environment remain poorly understood. We link natural variation in the thermotolerance of neural function and behavior in *Drosophila melanogaster* to the *foraging* gene (*for,* which encodes a cGMP-dependent protein kinase (PKG)) as well as to its downstream target, protein phosphatase 2A (PP2A). Genetic and pharmacological manipulations revealed that reduced PKG (or PP2A) activity caused increased thermotolerance of synaptic transmission at the larval neuromuscular junction. Like synaptic transmission, feeding movements were preserved at higher temperatures in larvae with lower PKG levels. In a comparative assay, pharmacological manipulations altering thermotolerance in a central circuit of *Locusta migratoria* demonstrated conservation of this neuroprotective pathway. In this circuit, either the inhibition of PKG or PP2A induced robust thermotolerance of neural function. We suggest that PKG and therefore the polymorphism associated with the allelic variation in *for* may provide populations with natural variation in heat stress tolerance. *for*'s function in behavior is conserved across most organisms, including ants, bees, nematodes, and mammals. PKG's role in thermotolerance may also apply to these and other species. Natural variation in thermotolerance arising from genes involved in the PKG pathway could impact the evolution of thermotolerance in natural populations.

## Introduction

Exposure to extreme ambient temperatures will result in the eventual failure of normal neural functioning. This is most evident in poikilothermic organisms that have evolved numerous adaptations to temperature stress [1], [2] including cellular homeoviscosity [3], heat shock responses [4], evaporative heat loss [5] and a suite of behavioral strategies [6]. At extremely high but sub-lethal temperatures, neural failure occurs through a string of events that start with motor pattern arrhythmicity which leads to spreading depression and eventual synaptic transmission failure. In humans, high internal body temperatures can lead to seizures, respiratory distress, cognitive dysfunction, brain damage or death [7], [8]. Because neural output failure occurs before permanent thermal damage [9], there exists a potential for the recovery of circuit function upon return to normal temperatures. Yet, little is known about the substrates responsible for thermotolerance of the nervous system to stresses such as hyperthermia. Here we investigate natural variation in endogenous protection mechanisms exhibited by insects and discover a novel pathway that acts to increase the thermotolerance of the nervous system.

In nature, *Drosophila melanogaster* larvae spend their lives feeding and moving through fermenting fruit which can reach temperatures ranging from 10–50°C [10]. Natural allelic variation in the *foraging (for)* gene, which encodes a cGMP-dependent protein kinase (PKG) results in rover (*for^R^*) or sitter (*for^s^*) larval foraging behaviors [11]. Rovers move more than sitters when feeding, and have higher PKG transcript levels and activities [12]. However, it remains unclear how levels of PKG modulate neural function to influence behavior. Here we demonstrate for the first time that natural allelic variation in *for* influences levels of heat stress tolerance.

Two separate findings suggested a potential relationship between *for*-PKG and thermotolerance of neural function during hyperthermia [13], [14]. First, reduced PKG activity in sitters is associated with more transient (less conductance) neuronal K^+^ currents than rovers, and pharmacological inhibition of PKG substantially reduces K^+^ conductances [13]. Second, in locusts, similar transient and reduced K^+^ currents have been associated with heat shock-mediated protection of neural function, including induced thermotolerance of synaptic transmission [14] and central pattern generation [4]. Thus, our hypothesis is that PKG, as a regulator of neuronal thermotolerance, could mediate protection against heat-induced neural trauma in *Drosophila melanogaster* and *Locusta migratoria*. Our results suggest a conserved function of the PKG pathway in thermotolerance.

## Results and Discussion

### Thermotolerance of behavior correlates with reduction of PKG

**Figure 1:**
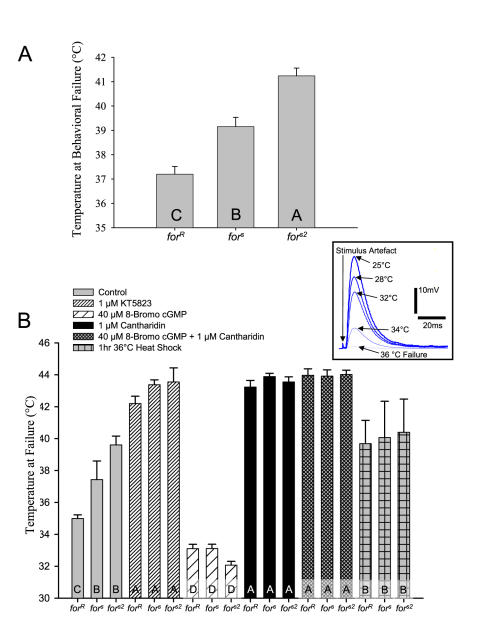
Hyperthermic failure of both behavior and NMJ synaptic transmission in 3^rd^ instar *for^R^*, *for^s^* and *for^s2^ Drosophila melanogaster* larvae. (A) Temperature at behavioral failure of mouth hook movement significantly differed between larvae with different *for* genotypes, *for^R^* failed at 37.2°C±0.3 (N = 30), *for^s^* failed at 39.2°C±10.4 (N = 30) and *for^s2^* failed at 41.2°C±0.3 (N = 30). Significant differences were found across groups (Kruskal-Wallis on ranks, H_(2,90)_ = 37.617, p<0.001) where letters (A, B, C) denote significant differences using a post-hoc test (Tukey, *p*<0.05). (B) Hyperthermic failure of evoked excitatory junction potential (EJP) failure (see inset) at the NMJ N>5 for all genotype and treatment combinations. Decreased thermotolerance of evoked synaptic transmission correlated with genotype (*for^s2^*>*for^s^*>*for^R^* for thermotolerance), where significant differences were found across treatment groups (Two Way ANOVA, F_(5,118)_ = 175.20, p<0.001). The involvement of PKG activity in thermotolerance was confirmed using pharmacological agents to activate PKG (40 µM 8-Bromo cGMP), inhibit PKG (1 µM KT5823) or inhibit a PKG phosphorylation target PP2A (1 µM Cantharidin). A combination of 8-Bromo cGMP and Cantharidin was also used, demonstrating that Cantharidin likely acts downstream of PKG activation. The three genotypes did not differ after being treated with a prior heat shock of 36°C for 1 hour and a 30 minute recovery. Letters in histogram bars represent statistical groupings using a post-hoc test, whereby bars with different letters are significantly different (Tukey, *p*<0.05). Error bars represent SEM.

To determine if *Drosophila for* variants have different levels of thermotolerance, we developed a behavioral assay that allowed us to increase temperature systematically and record the temperature at which larval mouth hook movements failed. Larval mouth hooks are critical for growth and survival because they are used to feed and move [15]. Mouth hook movements are easily visible in our preparation (see methods). When we increased temperature linearly at 5°C/min (starting from 22°C) the natural rover variant, *for^R^*, exhibited mouth hook movement failure (see methods) at a significantly lower temperature than the natural sitter *for^s^* (∼2°C lower; Figure 1A). Similarly, *for^s2^*, the sitter mutant generated on a rover genetic background exhibited significantly higher failure temperatures than *for^s^* and *for^R^* suggesting that sitters with their lower PKG levels have increased thermotolerance [11]. Finally, because the *for^R^* and *for^s2^* strains share a common genetic background, our results demonstrate that the rover/sitter differences in thermotolerance are specific and localizable to *for*.

### Thermotolerance of synaptic transmission via reduced PKG or PP2A inhibition

To investigate the neural underpinnings of differences in thermotolerance between the *for* variants, we assayed evoked excitatory junction potentials (EJPs) at larval muscle 6. We increased temperature at a rate of 5°C/min (starting from 22°C) and found that synaptic transmission in *for^s^* and *for^s2^* failed (response less than 1 mV) at significantly higher temperatures than in *for^R^*, suggesting that lower PKG activity leads to increased thermoprotection (Figure 1B). In order to confirm this, we used pharmacological manipulations of the PKG pathway. We pretreated dissected larval preparations with combinations of the cell-permeable PKG-specific inhibitor KT5823 and the PKG activator 8-Bromo-cGMP. Pharmacological inhibition of PKG significantly increased the temperature of synaptic failure in *for^R^*, *for^s^*, and *for^s2^* larvae; in this case, failure for all three strains was not observed until ∼42°C (Figure 1B). In contrast, activation of PKG via 8-bromo-cGMP significantly decreased thermotolerance (failure was observed at ∼33°C) of synaptic transmission compared to non-treated controls in all *for* strains (Figure 1B).

To explore what might act downstream of PKG in thermotolerance, we looked for potential candidate molecules known to be intermediaries of both PKG and K^+^ channels. Interestingly, PKG is known to phosphorylate protein phosphatase 2A (PP2A) leading to the de-phosphorylation of specific K^+^ channels and an increase in channel conductance [16], [17]. We found that the PP2A-specific inhibitor Cantharidin increased the thermotolerance of synaptic transmission as strongly as did the PKG inhibitor (Figure 1B). To test if PP2A inhibition acted within the PKG pathway we simultaneously applied both the PKG activator (8-Bromo-cGMP) and the PP2A inhibitor (Cantharidin) to the preparation. We found that the decrease in thermotolerance found by increasing PKG activity with 8-Bromo-cGMP was abolished when PP2A was inhibited, suggesting PP2A acts downstream of PKG. Thus, both genetic and pharmacological analyses demonstrate that there is a negative relationship between PKG activity and the thermotolerance of neuromuscular transmission in *D. melanogaster* larvae. These results parallel those found for mouth hook movements (Figure 1A), our behavioral measure of thermotolerance.

### PKG inhibition and PP2A inhibition induce rapid thermotolerance of neural circuitry

**Figure 2:**
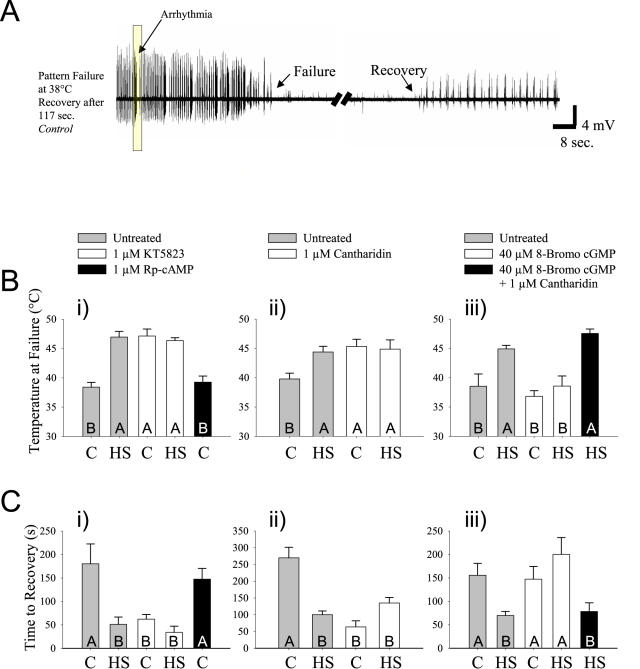
Hyperthermic failure of locust ventilatory motor pattern generation. (A) Sample traces of the ventilatory rhythm recorded from an abdominal expiratory muscle in a control locust. Note the ventilatory arrhythmias prior to failure. At failure, temperature was allowed to return to room temperature and time to recovery was recorded. Note that the ventilatory motor pattern after recovery is at a lower frequency just prior to failure because the temperature is lower. (B) Three separate experiments were performed to examine the pharmacological effects of i) PKG and PKA inhibitors ii) PP2A inhibitor and iii) PKG activator and PKG activator coupled with the PP2A inhibitor. (B i) Reduction in PKG activity (1 µM KT5823) increased the thermotolerance of neural function as strongly as a prior heat shock treatment during hyperthermia, whereas PKA inhibition (1 µM Rp-cAMP) had no effect. Significant treatment effects were found (ANOVA F_(4,31)_ = 18.71, p<0.001; N>4 for all treatments) where letters (A, B, C) denote significant differences using a post-hoc test (Tukey, *p*<0.05). (B ii) Reduction of PP2A activity (1 µM Cantharidin) also increased the thermotolerance of neural function during hyperthermia where significant treatment effects were found (ANOVA F_(3,31)_ = 4.60, p<0.001; N>6 for all treatments). (B iii) Activation of PKG using 40 µM 8-Bromo cGMP did not increase the thermotolerance of the circuit, but abolished the protective effects of heat shock preconditioning. Moreover, the PP2A inhibitor Cantharidin counteracted the effect of 8-Bromo cGMP in HS animals thereby inducing maximal thermotolerance. Significant treatment effects were found (ANOVA F_(4,41)_ = 9.60, p<0.001; N>6 for all treatments). (C) Recovery time of the motor pattern upon return to room temperature showed corresponding differences in the same treatment groups (short recovery times associated with high failure temperatures in B). Here too significant differences were found across treatments: i) ANOVA F_(4,31)_ = 7.62, p<0.001, N>4; ii) ANOVA F_(3,32)_ = 19.65, p<0.001, N>6; iii) ANOVA F_(4,41)_ = 4.78, p<0.001, N>6, Letters in histogram bars represent statistical groupings whereby bars with different letters are significantly different using a post-hoc test (Tukey, *p*<0.05). Error bars represent SEM.

To determine if the thermoprotective consequences of PKG manipulations are conserved and also apply to central circuitry and motor pattern generation, we measured the effects of PKG manipulation in an established model system used to study thermotolerance, the ventilatory motor pattern generator of the locust, *Locusta migratoria* [4]. Locusts that have undergone a prior heat shock exhibit a robust ∼10°C increase to the thermotolerance of neural function during hyperthermia [4], [18] as well as a reduction in neuronal whole cell potassium currents [14]. This suggests that PKG-mediated K^+^ current reductions in this model could cause significant changes in thermal protection. We observed thermoprotection and rapid recovery of the circuit when we pressure-injected KT5823 into the neural circuit for ventilation ten minutes prior to increasing the temperature (Figure 2B). Our initial trials of the induction of thermotolerance in this circuit via PKG inhibition demonstrated magnitudes of response equal to the data on previously published heat shock-pretreated locusts [4], [18]. Thus, for comparison, we examined neural output failure (observable loss of firing pattern >2 sec) from both control (C, no heat shock) and heat-shocked (HS) locusts with a combination of pharmacological treatments similar to those used in the *Drosophila* preparation [combinations of cell-permeable PKG-specific inhibitor (KT5823), PKG activator (8-Bromo-cGMP), PP2A inhibitor (Cantharidin)].

In three separate experiments, a prior heat shock induced a significant increase in thermotolerance of the ventilatory motor pattern generator increasing the tolerance of the circuit by ∼8°C (Figure 2B i–iii). Untreated controls (no pharmacological manipulation, no heat shock) also took approximately twice the time for the circuit to recover when compared to untreated heat shocked animals (Figure 2C i–iii). These results are similar to prior reports on the effect of a HS pretreatment on central circuitry during hyperthermia [4]. Thermotolerance was significantly increased in control animals by the application of the PKG inhibitor KT5823 (Figure 2Bi) and the PP2A inhibitor Cantharidin (Figure 2Bii), extending the thermotolerance of the neural circuit by ∼8°C. Further, recovery time of the circuit was also significantly decreased (∼70%) in KT5823-treated animals (Figure 2Ci) and Cantharidin-treated animals (Figure 2Cii) compared with untreated controls. Interestingly, there were no significant differences between untreated HS preparations and animals treated with either PKG or PP2A inhibitors except for the recovery time of Cantharidin-treated HS animals. Therefore, PKG or PP2A inhibition increases the thermotolerance of neural function in this locust central circuit and this response was as equally pronounced as a prior conditioning heat shock. We found the same pattern in the thermotolerance of synaptic transmission at the NMJ of *Drosophila* larvae. *for^R^*, *for^s^*, and *for^s2^* did not differ in thermotolerance when exposed to a prior heat shock of 36°C for 1 hr with a 30 min recovery (Figure 1B).

In order to determine if the observed thermoprotective effects are specific to the inhibition of PKG, we examined the effects of inhibition of protein kinase A (PKA) using Rp-cAMP. Rp-cAMP produced no significant differences in failure temperature or recovery time compared to untreated controls (Figures 2Bi,Ci). Interestingly, previous work has shown that PKA influences thermotolerance of neural function in an opposite fashion to PKG and in a much slower time scale (1 hr vs. several min) [19].

To examine the rapidity of the effect of pharmacological manipulation on thermotolerance we developed an acute treatment with the PKG inhibitor KT5823. When the temperature ramp reached 30°C (approximately three minutes before failure), we pressure-injected 25 nL of 1 µM KT5823 into previously untreated animals and observed an extremely rapid and equally pronounced thermal protection of the circuit when compared to data shown in Figure 2A (not shown). This showed that unlike a heat shock conditioning, the protective effect of PKG and PP2A inhibition can be induced rapidly.

### PKG activator 8-bromo-cGMP abolishes thermoprotective effect of a prior heat shock

To obtain a better understanding of how the protective effect of a heat shock pre-treatment interacts with the PKG pathway, we examined the effect of the PKG activator 8-Bromo cGMP on previously heat shocked locusts. Initially we found that there was no significant difference in failure temperature or recovery time of the circuit in non-heat shocked animals with and without 8-Bromo cGMP (Figures 2B, C). However, HS-mediated thermotolerance was abolished with the addition of 8-Bromo cGMP. Compared with untreated HS animals, simultaneous applications of both the PKG activator and the PP2A inhibitor in HS animals resulted in maximal thermotolerance in failure temperature and reduced times to recovery. These results coupled with the *Drosophila* larval experiments (see above) indicated that PP2A likely acts downstream from PKG and interacts with the same pathway affected by heat shock preconditioning.

### PKG and PP2A inhibition abolish arrhythmic events arising from heat stress

Arrhythmias (pattern interruptions) in the ventilatory motor pattern indicate stress-induced transient malfunction of the pattern generating mechanisms. We found that either a prior heat shock or inhibition of PKG or PP2A reduced the number of arrhythmias observed during hyperthermia (not shown). Locusts that did not receive a pharmacological treatment or a heat shock had the highest prevalence of arrhythmias observed across all preparations during temperature increases (60%). However, 8-bromo-cGMP-treated control (50%) and 8-bromo-cGMP-treated heat shock (40%) preparations were also high compared to all other treatments. Finally, non-heat shocked animals exposed to PKG inhibitor exhibited the lowest occurrence of arrhythmias (12.5%). Thus, both PKG and PP2A inhibition not only protect synaptic transmission between synapses, but their inhibition also protects the delicate coordination of neural circuitry during hyperthermic stress. Inhibition of these enzymes did not alter neural temporal properties.

Little is known about the genes and pathways that contribute to nervous system function under thermal stress despite their obvious importance to the survival of organisms. We identify two molecules, cGMP-dependent protein kinase (PKG) and protein phosphatase 2A (PP2A) that rapidly and dramatically extend the thermal operating range of neural function. By decreasing PKG or PP2A activity we increased the thermotolerance of synaptic transmission at the NMJ of *Drosophila* larvae and the thermotolerance of the ventilatory motor pattern in adult *Locusta*. Similarly, activation of PKG via 8-Bromo cGMP decreased the thermotolerance of synaptic transmission at the NMJ of *Drosophila* larvae and abolished the thermotolerance conferred via a prior heat shock in adult *Locusta*.

Surprisingly, in spite of the numerous investigations into the roles of HSPs, little is known about their mechanisms of action in nervous tissue. Specifically, it is not known whether signaling pathways such as PKG and PP2A act in conjunction with the HS response pathway to protect neural function [18], [20]. However, as mentioned previously, our ability to abolish the protective effects of a prior HS through the application of cGMP in *Locusta* suggests that PKG may interact with the HS response pathway. One possible model is that PKG-PP2A interacts with a stress inducible heat shock protein such as HSP27. HSP27 is a chaperone from the small HSP family known to confer protection from starvation, hyperthermia and oxidative stress and to play a role in *Drosophila* lifespan [21]. In order for HSP27 to act as a chaperone, it requires dephosphorylation by PP2A [22]. We have shown pharmacologically that a decrease in PP2A rapidly induces thermotolerance of neural function (Figures 1&2). Thus, it is feasible that the previously reported slower time course (3 to 4 hr) of protection to neural function resulting from a prior HS [4], [14], [18], [23] may be due to upregulated HSP27 which may use a large proportion of the available active intracellular PP2A, thereby mimicking PP2A inhibition. Whether PKG-PP2A directly interacts with heat shock response pathways remains to be determined.

The hyperthermic temperatures used in our experiments are ecologically relevant. Fermenting fruit, a main habitat of *D. melanogaster* larvae, can range in temperature from 10–50°C in nature and temperatures at the higher end are lethal for *D. melanogaster* larvae [10]. Thus, the rover/sitter differences in thermotolerance and behavior should have fitness consequences in nature. Sitter larvae are expected to have higher survivorship while foraging in fruit under hyperthermic conditions. In fact, *D. melanogaster* larvae collected from a desert site in Tunisia behaved as sitters whereas those collected 1500 m away in an oasis site behaved more rover-like [24]. Rovers may avoid temperature stress within the fruit because of their greater tendency to leave their food [25]. To what extent the observed ∼2°C differences in the upper temperature limit for larval mouth hook movement behavior in rovers and sitters reflects fitness differences in nature remains to be determined. Interestingly, a 2°C increase in seasonal environmental temperatures in the poikilothermic common eelpout, *Zoarces viviparous,* has dramatic effects on survival in the wild [26]. Similarly, the survivorship of rover and sitter larvae in nature could vary depending on how hyperthermic conditions vary in space and time.

We found similar functions for PKG and PP2A in rapid thermoprotection of the nervous systems of both *D. melanogaster* and *L. migratoria*. This suggests a conserved role for these molecules in thermotolerance in response to environmental stress. Whether or not our findings can be extended to mammalian models remains to be determined. If so, manipulations of the PKG pathway could be used to rapidly treat neural failure during hyperthermic and febrile episodes in mammals, including humans.

## Materials and Methods

### *D. melanogaster* Experiments

#### Animals

We used foraging 3^rd^ instar (96±2 hr) *Drosophila melanogaster* larvae reared on a yeast-sugar-agar medium at 24±1°C under 12L:12D light cycle with lights on at 0800 hr. We used a rover strain homozygous for the *for^R^* allele, and two sitter strains homozygous for *for^s^* and *for^s2^*. The wild type-derived rover (*for^R^*) and sitter (*for^s^*) strains are natural allelic variants of the *for* gene which is located on chromosome-2. To control for genetic background they have co-isogenic third chromosomes (originating from the rover strain) and shared X-chromosomes. The *for^s2^* strain is a sitter mutant generated on a rover *for^R^* genetic background [27] such that *for^s2^* differs from *for^R^* only in their alleles at *for. for^s^* and *for^s2^* larvae have significantly lower PKG enzyme activity than *for^R^* [11]. N indicates the number of animals while n indicates the number of trials,. Both are reported in the figure legends.

#### Behavior

*Drosophila* larvae were secured to a glass dish in 2 ml of Schneider's Insect Medium (Sigma) using a blunt magnetic placed pin distal to the CNS. Preparations were heated on a Peltier plate (5°C/min) from 22°C. We measured the persistence of rhythmic mouth hook movement. The frequency of mouth hook movements increased (>3 Hz) with increasing temperature until abrupt failure. Failure of mouth hook movement was considered to have occurred when mouth hook movements stopped for at least 30 sec. The temperature at the beginning of this 30 sec period was recorded as the failure temperature. Pauses in mouth hook movement occur occasionally although no difference in pausing was observed between rovers and sitters. The temperature was controlled by a single switch (on/off) and no other settings are manipulated. Prior to each experiment the ramping of temperature was recorded for several runs to verify its accuracy. We found it to be highly consistent.

#### Electrophysiology

*Drosophila* larvae were dissected and pinned on a glass dish in Schneider's Insect Medium (Sigma); internal organs and the central nervous systems were removed to reveal the segmental muscles and their corresponding nerves. Segmental nerves in segments 3 or 4 were stimulated using a suction electrode and the corresponding excitatory junction potential (EJP) was recorded from muscle 6 with a glass intracellular electrode filled with 3 M KCl (40–80 MΩ). The preparation was superfused with HL-6 saline [28] with 1 mM [Ca^2+^]_e_ and heated at a rate of approximately 5°C/min from room temperature (∼20°C) to synaptic failure (Figure 1B inset). Pharmacological treatments of preparations included one or a combination of the following agents (all chemicals obtained from Sigma): 1 µM KT5823 (Calbiochem; PKG-specific inhibitor; a relatively specific PKG inhibitor [29] that competes for the ATP binding site on the kinase [30]), 40 µM 8-bromo-guanosine 3′,5′-cyclic monophosphate (VWR; 8-bromo-cGMP; cGMP analogue), 1 µM Cantharidin (Calbiochem; PP2A-specific inhibitor; a cell permeable terpenoid that binds to PP2A at the “Cantharidin binding site” to inhibit its action [31]) and 1 µM Rp-cAMP (PKA inhibitor; competitive with cAMP at PKA binding sites[32]). Preparations were bathed with the compound(s) of interest for 5 min prior to heating. KT5823, 8-Bromo-cGMP, and Cantharidin were dissolved in DMSO, resulting in a 0.2% (v/v) DMSO concentration in the saline during treatment. 0.2% (v/v) DMSO in physiological saline was used as a control for the solvent's effects on synaptic transmission, and no differences were observed.

Cantharidin (Specificity: PP2A>PP1≫PP2B) is an inhibitor of protein phosphatase 2A (IC_50_ = 40 nM) and it can inhibit protein phosphatase 1 at higher concentrations (IC_50_ = 473 nM). To rule out possible effects of protein phosphatase 1 inhibition, we measured the temperature of failure of synaptic transmission in *for^R^* at the NMJ using a low 100 nM concentration of Cantharidin [33], [31], [34]. This concentration is known to specifically inhibit PP2A. We found that synaptic transmission at the NMJ in *for^R^* treated with 100 nM Cantharidin failed at 42.7°C+/−0.33, whereas *for^R^* treated with 1 µM Cantharidin failed at 43.2°C+/−0.42; no significant differences were found between the high and low Cantharidin treatments (Student's t-test, t = 1.045, df = 11, P = 0.319).

### *L. migratoria* Experiments

#### Animals

Male locusts aged 4–6 weeks were obtained from a crowded colony (12:12 photoperiod; 25±1°C daytime temperature) maintained in the Department of Biology at Queen's University. Heat shocked (HS) animals were held for 3 hr in a 2 L plastic container in a humid incubator at 45°C and allowed 1 hr for recovery. Control animals were held at room temperature for 4 hr prior to experiments. Animals were dissected and pinned on a cork board; overlying tissue was removed exposing the dorsal surface of the metathoracic ganglion.

#### Electrophysiology

Ventilatory motor patterns were monitored using a copper electromyographic (EMG) electrode positioned on expiratory muscle 161 in the second abdominal segment. Standard locust saline was superfused through thoracic and abdominal cavities and heated at a rate of 5°C/min until motor pattern failure at which point saline was allowed to return to room temperature. Pressure injections (32±13.5 nL, 10 psi, 150 ms) into the ventilatory neuropil were made with glass microelectrodes filled with either 1 µM KT5823 (using DMSO) in standard locust saline or locust saline alone (sham pressure-injections contained DMSO) 10 min before the temperature ramp (pre-treated) or during the temperature ramp at 30°C (acutely treated; ∼3 min before failure in controls).

### Statistics

Statistical analyses used One-Way and Two-Way ANOVA (F is reported) and One-Way ANOVA on ranks (H is reported; Kruskal Wallis) followed by a post-hoc Tukey Multiple Comparison test. Figures show significant differences using letter designations, where A is the highest mean, B indicates the next highest mean, etc. Data points with different letters show significant differences (P<0.05).
